# Supplementary material for: Phylogenetic and Haplotype Network Analyses of Diaporthe eres Species in China Based on Sequences of Multiple Loci
Source: Biology (Basel). 2021 Mar 1;10(3):179. doi: 10.3390/biology10030179 (PMC8000818; doi:10.3390/biology10030179)
Supplement: Supplementary file 1 [file biology-10-00179-s001.pdf]

Supplementary Material

Phylogenetic and Haplotype Network of *Diaporthe eres* Species in China Based on Analyses of Multiple Loci

Chingchai Chaisiri [1,2,3], Xiangyu Liu [1,2,3], Yang Lin [2,3], Yanping Fu [2,3], Fuxing Zhu [3] and Chaoxi Luo [1,2,3,*]

[1] Key Lab of Horticultural Plant Biology, Ministry of Education, Huazhong Agricultural University, Wuhan 430070, China; chaisiri.ch@gmail.com (C.C.); xiangyuliu@webmail.hzau.edu.cn (X.L.)
[2] Key Lab of Crop Disease Monitoring and Safety Control in Hubei Province, Huazhong Agricultural University, Wuhan 430070, China; yanglin@mail.hzau.edu.cn (Y.L.); yanpingfu@mail.hzau.edu.cn (Y.F)
[3] College of Plant Science and Technology, Huazhong Agricultural University, Wuhan 430070, China; zhufuxing@mail.hzau.edu.cn (F.Z.)
* Correspondence: cxluo@mail.hzau.edu.cn (C.L.)

Table S1. List of *Diaporthe eres* isolates used for population analysis, with details about origin, host, isolate number, and GenBank accession number.

Origin	Host	Isolate number	GenBank accession number [a]					Reference(s)
			ITS	*EF-1α*	*TUB2*	*CAL*	*HIS*	
Beijing	*Castanea mollissima*	CFCC 52576	MH121511	MH121553	MH121593	MH121432	MH121471	[1]
	Castanea mollissima	CFCC 52577	MH121512	MH121554	MH121594	MH121433	MH121472	[1]
	Juglans regia	CFCC 52005	MF279855	MF279870	MF279885	MF279899	MF279840	[2]
	Juglans regia	CFCC 52006	MF279856	MF279871	MF279886	MF279900	MF279841	[2]
	Juglans regia	CFCC 52009	MF279852	MF279867	MF279882	MF279896	MF279837	[2]
	Juglans regia	CFCC 52010	MF279853	MF279868	MF279883	MF279897	MF279838	[2]
	Juglans regia	CFCC 52015	MF279848	MF279863	MF279878	MF279892	MF279833	[2]
	Juglans regia	CFCC 52016	MF279849	MF279864	MF279879	MF279893	MF279834	[2]
	Vitis sp.	ICMP 20397	KJ609009	KJ623309	KJ623295	KJ623277	–	[3]
	Vitis sp.	ICMP 20398	KJ609010	KJ623308	KJ623294	KJ623276	–	[3]
	Vitis sp.	ICMP 20399	KJ609011	KJ623307	KJ623293	KJ623275	–	[3]
Chongqing	*Pyrus pyrifolia*	PSCG 245	MK626894	MK654822	MK691274	MK691164	MK726224	[4]
	Pyrus pyrifolia	PSCG 250	MK626895	MK654836	MK691275	MK691168	MK726245	[4]
Fujian	*Pyrus pyrifolia*	PSCG 132	MK626891	MK654816	MK691250	MK691133	MK726212	[4]
	Pyrus pyrifolia	PSCG 135	MK626873	MK654837	MK691251	MK691160	MK726213	[4]

Origin	Host	Isolate number	GenBank accession number [a]					Reference(s)
			ITS	*EF-1α*	*TUB2*	*CAL*	*HIS*	
	Pyrus pyrifolia	PSCG 151	MK626876	MK654820	MK691262	MK691161	MK726239	[4]
Gansu	*Juglans regia*	CFCC 52007	MF279857	MF279872	MF279887	MF279901	MF279842	[2]
	Juglans regia	CFCC 52008	MF279854	MF279869	MF279884	MF279898	MF279839	[2]
Hebei	*Vitis* sp.	ICMP 20400	KJ609012	KJ623306	KJ623292	KJ623274	–	[3]
	Vitis sp.	ICMP 20402	KJ609006	KJ623312	KJ623285	KJ623280	–	[3]
Henan	*Juglans regia*	CFCC 52003	MF279845	MF279860	MF279875	–	MF279830	[2]
	Pyrus pyrifolia	PSCG 321	MK626874	MK654827	MK691267	MK691167	MK726228	[4]
	Pyrus pyrifolia	PSCG 322	MK626875	MK654824	MK691268	MK691162	MK726224	[4]
	Pyrus pyrifolia	PSCG 324	MK626906	MK654830	MK691272	MK691149	MK726220	[4]
	Pyrus pyrifolia	PSCG 325	MK626905	MK654838	MK691273	MK691153	MK726222	[4]
	Pyrus pyrifolia	PSCG 346	MK626882	MK654848	MK691270	MK691134	MK726234	[4]
Hubei	*Prunus persica*	MFLUCC 16-0097	KU557547	KU557615	KU557571	KU557595	–	[5]
	Prunus persica	MFLUCC 16-0098	KU557548	KU557616	KU557572	KU557596	–	[5]
	Prunus persica	MFLUCC 16-0099	KU557549	KU557617	KU557573	KU557597	–	[5]
	Prunus persica	MFLUCC 16-0100	KU557550	KU557618	KU557574	KU557598	–	[5]
	Prunus persica	MFLUCC 16-0101	KU557551	KU557619	KU557575	KU557599	–	[5]
	Prunus persica	MFLUCC 16-0102	KU557552	KU557620	KU557576	KU557600	–	[5]
	Prunus persica	MFLUCC 16-0103	KU557553	KU557621	KU557577	KU557601	–	[5]
	Prunus persica	MFLUCC 16-0104	KU557554	KU557622	KU557578	KU557602	–	[5]
	Prunus persica	MFLUCC 16-0109	KU557559	KU557627	KU557583	KU557607	–	[5]
	Prunus persica	MFLUCC 16-0110	KU557560	KU557628	KU557584	KU557608	–	[5]
	Prunus persica	MFLUCC 16-0111	KU557561	KU557629	KU557585	KU557609	–	[5]
	Prunus persica	MFLUCC 16-0112	KU557562	KU557630	KU557586	KU557610	–	[5]
	Prunus persica	MFLUCC 16-0113	KU557563	KU557631	KU557587	KU557611	–	[5]
	Prunus persica	MFLUCC 16-0114	KU557564	KU557632	KU557588	KU557612	–	[5]
	Prunus persica	MFLUCC 16-0115	KU557565	KU557633	KU557589	KU557613	–	[5]
	Prunus persica	MFLUCC 16-0116	KU557566	KU557634	KU557590	KU557614	–	[5]
	Pyrus pyrifolia	PSCG 261	MK626904	MK654826	MK691252	MK691141	MK726241	[4]
	Pyrus pyrifolia	PSCG 265	MK626903	MK654842	MK691282	MK691150	MK726214	[4]
	Pyrus pyrifolia	PSCG 299	MK626900	MK654818	MK691255	MK691154	MK726246	[4]
	Pyrus pyrifolia	PSCG 300	MK626901	MK654819	MK691253	MK691155	MK726247	[4]
	Pyrus pyrifolia	PSCG 440	MK626908	MK654825	MK691256	MK691140	MK726230	[4]
	Vitis vinifera	JZB320033	MK335719	MK523622	MK500179	MK500072	–	[6]

Origin	Host	Isolate number	GenBank accession number [a]					Reference(s)
			ITS	*EF-1α*	*TUB2*	*CAL*	*HIS*	
	Vitis vinifera	JZB320034	MK335720	MK523623	MK500180	MK500073	–	[6]
	Vitis vinifera	JZB320035	MK335721	MK523593	MK500181	MK500074	–	[6]
	Vitis vinifera	JZB320036	MK335722	–	MK500182	MK500075	–	[6]
	Vitis vinifera	JZB320037	MK335723	–	MK500183	MK500076	–	[6]
	Vitis vinifera	JZB320038	MK335724	MK523594	MK500184	MK500077	–	[6]
	Vitis vinifera	JZB320039	MK335725	MK523595	MK500185	MK500078	–	[6]
	Vitis vinifera	JZB320040	MK335726	MK523596	MK500186	MK500079	–	[6]
	Vitis vinifera	JZB320041	MK335727	–	MK500187	MK500080	–	[6]
	Vitis vinifera	JZB320043	MK335728	MK523624	MK500188	MK500081	–	[6]
	Vitis vinifera	JZB320044	MK335729	–	MK500189	MK500082	–	[6]
	Vitis vinifera	JZB320045	MK335730	MK523597	–	MK500083	–	[6]
	Vitis vinifera	JZB320046	MK335731	MK523598	MK500190	MK500084	–	[6]
	Vitis vinifera	JZB320047	MK335732	–	MK500191	MK500085	–	[6]
	Vitis vinifera	JZB320048	MK335733	MK523599	MK500192	MK500086	–	[6]
	Vitis vinifera	JZB320049	MK335734	MK523625	MK500193	MK500087	–	[6]
	Vitis vinifera	JZB320051	MK335735	MK523600	MK500194	MK500088	–	[6]
	Vitis vinifera	JZB320052	MK335736	–	MK500195	MK500089	–	[6]
Jiangsu	*Camptotheca acuminata*	CFCC 51632	KY203726	KY228887	KY228893	KY228877	KY228881	[7]
	Camptotheca acuminata	CFCC 51633	KY203727	KY228888	KY228894	KY228878	KY228882	[7]
	Pyrus pyrifolia	PSCG 175	MK626877	MK654843	MK691259	MK691165	MK726238	[4]
	Pyrus pyrifolia	PSCG 512	MK626883	MK654832	MK691271	MK691135	MK726240	[4]
	Pyrus pyrifolia	PSCG 521	MK626888	MK654850	MK691284	MK691136	MK726233	[4]
	Pyrus pyrifolia	PSCG 529	MK626902	MK654831	MK691258	MK691156	MK726242	[4]
Jiangxi	*Citrus reticulata* cv. Nanfengmiju	NFFL-1-25	**MN816412**	**MN894433**	**MN894472**	**–**	**MN894395**	[8]
	Citrus reticulata cv. Nanfengmiju	NFFL-1-36	**MN816413**	**MN894434**	**MN894473**	**MN894365**	**MN894396**	[8]
	Citrus reticulata cv. Nanfengmiju	NFFL-2-17	**MN816414**	**MN894435**	**MN894474**	**–**	**MN894397**	[8]
	Citrus reticulata cv. Nanfengmiju	NFFL-2-8	**MN816415**	**MN894436**	**MN894475**	**MN894366**	**MN894398**	[8]
	Citrus reticulata cv. Nanfengmiju	NFFL-3-1	**MN816416**	**MN894437**	**MN894476**	**MN894367**	**MN894399**	[8]
	Citrus reticulata cv. Nanfengmiju	NFFL-4-5	**MN816417**	**MN894438**	**MN894477**	**MN894368**	**MN894400**	[8]
	Citrus reticulata cv. Nanfengmiju	NFFT-3-3	**MN816418**	**MN894439**	**MN894478**	***MW221706***	**MN894401**	[8]
	Citrus reticulata cv. Nanfengmiju	NFFT-3-8	**MN816419**	**MN894440**	**MN894479**	**MN894369**	**MN894402**	[8]
	Citrus reticulata cv. Nanfengmiju	NFIF-1-1	**MN816420**	**MN894441**	**MN894480**	**MN894370**	**MN894403**	[8]
	Citrus reticulata cv. Nanfengmiju	NFIF-1-7	**MN816421**	**MN894442**	**MN894481**	**–**	**MN894404**	[8]

Origin	Host	Isolate number	GenBank accession number [a]					Reference(s)
			ITS	*EF-1α*	*TUB2*	*CAL*	*HIS*	
	Citrus unshiu	ZJUD90	KJ490625	KJ490504	KJ490446	–	KJ490567	[9]
	Citrus sp.	ZJUD91	KJ490626	KJ490505	KJ490447	–	KJ490568	[9]
	Pyrus pyrifolia	PSCG 007	MK626884	MK654835	MK691278	MK691157	MK726216	[4]
	Pyrus pyrifolia	PSCG 017	MK626887	MK654829	MK691283	MK691139	MK726232	[4]
	Pyrus pyrifolia	PSCG 023	MK626878	MK654821	MK691269	MK691158	MK726217	[4]
	Pyrus pyrifolia	PSCG 381	MK626897	MK654847	MK691277	MK691148	MK726215	[4]
Jilin	*Vitis vinifera*	JZB320054	MK335738	MK523602	MK500197	MK500091	–	[6]
	Vitis vinifera	JZB320055	MK335739	MK523617	MK500198	MK500092	–	[6]
	Vitis vinifera	JZB320056	MK335740	MK523618	MK500199	MK500093	–	[6]
	Vitis vinifera	JZB320057	MK335741	MK523603	MK500200	MK500094	–	[6]
	Vitis vinifera	JZB320058	MK335742	MK523604	MK500201	MK500095	–	[6]
	Vitis vinifera	JZB320059	MK335743	MK523605	MK500202	MK500096	–	[6]
	Vitis vinifera	JZB320060	MK335744	MK523606	MK500203	MK500097	–	[6]
	Vitis vinifera	JZB320061	MK335745	MK523607	MK500204	MK500098	–	[6]
	Vitis vinifera	JZB320062	MK335746	MK523614	MK500205	MK500099	–	[6]
	Vitis vinifera	JZB320063	MK335747	MK523608	MK500206	MK500100	–	[6]
	Vitis vinifera	JZB320070	MK335754	MK523613	MK500213	–	–	[6]
Liaoning	*Pyrus ussuriensis*	PSCG 358	MK626889	MK654849	MK691260	MK691143	MK726231	[4]
	Pyrus pyrifolia	PSCG 362	MK626907	MK654846	MK691280	MK691151	MK726235	[4]
Ningxia	*Juglans regia*	CFCC 52011	MF279850	MF279865	MF279880	MF279894	MF279835	[2]
	Juglans regia	CFCC 52012	MF279851	MF279866	MF279881	MF279895	MF279836	[2]
Shandong	*Castaneae mollissimae*	DNP128	JF957786	KJ210561	KJ420801	KJ435040	KJ420852	[10,11]
	Pyrus communis	PSCG 090	MK626872	MK654828	MK691281	MK691159	MK726236	[4]
	Pyrus communis	PSCG 092	MK626896	MK654823	MK691264	MK691147	MK726227	[4]
	Pyrus communis	PSCG 202	MK626885	MK654817	MK691254	MK691166	MK726237	[4]
	Pyrus communis	PSCG 306	MK626898	MK654839	MK691279	MK691138	MK726243	[4]
	Vitis sp.	ICMP 20401	KJ609013	KJ623305	KJ623291	KJ623273	–	[3]
Sichuan	*Juglans regia*	CFCC 52013	MF279846	MF279861	MF279876	MF279890	MF279831	[2]
	Juglans regia	CFCC 52014	MF279847	MF279862	MF279877	MF279891	MF279832	[2]
	Vitis vinifera	JZB320020	–	MK523586	MK500169	MK500062	–	[6]
	Vitis vinifera	JZB320021	MK335710	MK523587	MK500170	MK500063	–	[6]
	Vitis vinifera	JZB320022	MK335711	MK523588	MK500171	MK500064	–	[6]
	Vitis vinifera	JZB320026	MK335714	MK523591	MK500174	MK500067	–	[6]

Origin	Host	Isolate number	GenBank accession number [a]					Reference(s)
			ITS	*EF-1α*	*TUB2*	*CAL*	*HIS*	
Yunnan	*Pyrus bretschneideri*	PSCG 041	MK626880	MK654840	MK691265	MK691144	MK726219	[4]
	Pyrus bretschneideri	PSCG 042	MK626881	MK654845	MK691285	MK691145	MK726225	[4]
	Pyrus bretschneideri	PSCG 043	MK626879	MK654844	MK691266	MK691146	MK726229	[4]
Zhejiang	*Camellia oleifera*	CNUCC 201907	MN216301	MN224674	MN227014	MN224553	MN224562	[12]
	Citrus sp.	ZJUD92	KJ490627	KJ490506	KJ490448	–	KJ490569	[9]
	Lithocarpus glabra	CGMCC3.17081	KF576282	KF576257	KF576306	–	–	[13]
	Lithocarpus glabra	CGMCC3.17082	KF576283	KF576258	KF576307	–	–	[13]
	Lithocarpus glabra	CGMCC3.17083	KF576284	KF576259	KF576308	–	–	[13]
	Lithocarpus glabra	CGMCC3.17089	KF576267	KF576242	KF576291	–	–	[13]
	Lithocarpus glabra	CGMCC3.17090	KF576268	KF576243	KF576292	–	–	[13]
	Lithocarpus glabra	CGMCC3.17091	KF576269	KF576244	KF576293	–	–	[13]
	Lithocarpus glabra	CGMCC3.15181	KC153096	KC153087	KF576312	–	–	[14]
	Lithocarpus glabra	CGMCC3.17084	KF576270	KF576245	KF576291	–	–	[13]
	Lithocarpus glabra	CGMCC3.17085	KF576271	KF576246	KF576292	–	–	[13]
	Lithocarpus glabra	CGMCC3.17086	KF576272	KF576247	KF576293	–	–	[13]
	Melia azedarace	CFCC 52578	MH121513	MH121555	MH121595	MH121434	MH121473	[1]
	Pyrus pyrifolia	PSCG 276	MK626909	MK654841	MK691263	MK691163	MK726226	[4]
	Pyrus pyrifolia	PSCG 376	MK626899	MK654834	MK691257	MK691142	MK726218	[4]
	Pyrus pyrifolia	PSCG 377	MK626886	MK654833	MK691276	MK691137	MK726221	[4]
	Rhododendron simsii	CFCC 52581	MH121516	MH121558	MH121597	–	MH121476	[1]
	Vitis sp.	ICMP 20391	KJ609004	KJ623314	KJ623298	KJ623282	–	[3]
	Vitis sp.	ICMP 20393	KJ609014	KJ623303	KJ623284	KJ623267	–	[3]
	Vitis sp.	ICMP 20394	KJ609015	KJ623302	KJ623290	KJ623272	–	[3]
	Unknown	CNUCC 201908	MN216303	MN227020	MN227019	MN224554	MN224562	[12]

[a] ITS: nuclear ribosomal internal transcribed spacer regions; *EF1-α*: translation elongation factor 1-α gene; *TUB2*: beta-tubulin gene; *CAL*: calmodulin gene; and *HIS*: histone-3 gene. Sequences generated in our laboratory are indicated in **bold**.

References:

1. Yang, Q.; Fan, X.L.; Guarnaccia, V.; Tian, C.M. High diversity of *Diaporthe* species associated with dieback diseases in China, with twelve new species described. *MycoKeys* **2018**, *39*, 97–149.
2. Fan, X.L.; Yang, Q.; Bezerra, J.D.P.; Alvarez, L.V.; Tian, C.M. *Diaporthe* from walnut tree (*Juglans regia*) in China, with insight of the *Diaporthe eres* complex. *Mycol. Progress* **2018**, *17*, 841–853.

3. Dissanayake, A.J.; Liu, M.; Zhang, W.; Chen, Z.; Udayanga, D.; Chukeatirote, E.; Li, X.H.; Yan, J.Y.; Hyde, K.D. Morphological and molecular characterisation of *Diaporthe* species associated with grapevine trunk disease in China. *Fungal Biol.* **2015**, *119*, 283–294.
4. Guo, Y.S.; Crous, P.W.; Bai, Q.; Fu, M.; Yang, M.M.; Wang, X.H.; Du, Y.M.; Hong, N.; Xu, W.X.; Wang, G.P. High diversity of *Diaporthe* species associated with pear shoot canker in China. *Persoonia* **2020**, *45*, 132–162.
5. Dissanayake, A.J.; Zhang, W.; Liu, M.; Hyde, K.D.; Zhao, W.S.; Li, X.H.; Yan, J.Y. *Diaporthe* species associated with peach tree dieback in Hubei, China. *Mycosphere* **2017**, *8*, 533–549.
6. Manawasighe, I.S.; Dissanayake, A.J.; Li, X.H.; Liu, M.; Wanasinghe, D.N.; Xu, J.P.; Zhao, W.S.; Zhang, W.; Zhou, Y.Y.; Hyde, K.D., *et al.* High genetic diversity and species complexity of *Diaporthe* associated with grapevine dieback in China. *Front. Microbiol.* **2019**, *10*, 1936.
7. Yang, Q.; Fan, X.L.; Du, Z.; Liang, Y.M.; Tian, C.M. *Diaporthe camptothecicola* sp. nov. on *Camptotheca acuminata* in China. *Mycotaxon* **2017**, *132*, 591–601.
8. Chaisiri, C.; Liu, X.Y.; Lin, Y.; Li, J.B.; Xiong, B.; Luo, C.X. Phylogenetic analysis and development of molecular tool for detection of *Diaporthe citri* causing melanose disease of citrus. *Plants* **2020**, *9*, 329.
9. Huang, F.; Udayanga, D.; Wang, X.H.; Hou, X.; Mei, X.F.; Fu, Y.S.; Hyde, K.D.; Li, H.Y. Endophytic *Diaporthe* associated with *Citrus*: a phylogenetic reassessment with seven new species from China. *Fungal Biol* **2015**, *119*, 331–347.
10. Udayanga, D.; Castlebury, L.A.; Rossman, A.Y.; Chukeatirote, E.; Hyde, K.D. Insights into the genus *Diaporthe*: phylogenetic species delimitation in the *D. eres* species complex. *Fungal Divers.* **2014**, *67*, 203–229.
11. Udayanga, D.; Liu, X.Z.; Crous, P.W.; McKenzie, E.H.C.; Chukeatirote, E.; Hyde, K.D. A multi-locus phylogenetic evaluation of *Diaporthe* (*Phomopsis*). *Fungal Divers.* **2012**, *56*, 157–171.
12. Zhou, H.; Hou, C.L. Three new species of *Diaporthe* from China based on morphological characters and DNA sequence data analyses. *Phytotaxa* **2019**, *422*, 157–174.
13. Gao, Y.H.; Su, Y.Y.; Sun, W.; Cai, L. *Diaporthe* species occurring on *Lithocarpus glabra* in China, with descriptions of five new species. *Fungal Biol.* **2015**, *119*, 295–309.
14. Gao, Y.H.; Sun, W.; Su, Y.Y.; Cai, L. Three new species of *Phomopsis* in Gutianshan nature reserve in China. *Mycol. Progress* **2014**, *13*, 111–121.

Table S2. The best-fit corresponding nucleotide substitution models used in BI analysis.

**Partitioned dataset** [a]	**Nucleotide model under AIC** [b]	**Implemented parameters in BI analysis**
EF1-α	GTR+G	nst=6, rates=gamma
CAL	GTR+G	nst=6, rates=gamma
TUB2	HKY+G	nst=2, rates=gamma
HIS	GTR+I+G	nst=6, rates=invgamma
ITS	GTR+I+G	nst=6, rates=invgamma
EF1-α+CAL	GTR+G	nst=6, rates=gamma
EF1-α+TUB2	HKY+G	nst=2, rates=gamma
EF1-α+HIS	GTR+G	nst=6, rates=gamma
CAL+TUB2	HKY+G	nst=2, rates=gamma
CAL+HIS	HKY+I+G	nst=2, rates=invgamma
TUB2+HIS	HKY+G	nst=2, rates=gamma
EF1-α+CAL+TUB2	HKY+G	nst=2, rates=gamma
EF1-α+CAL+HIS	GTR+I+G	nst=6, rates=invgamma
EF1-α+CAL+TUB2+HIS	GTR+G	nst=6, rates=gamma
EF1-α+CAL+TUB2+HIS+ITS	GTR+I+G	nst=6, rates=invgamma

[a] *EF1-α*: translation elongation factor 1-*α* gene; *CAL*: calmodulin gene; *TUB2*: beta-tubulin 2 gene; *HIS*: histone-3 gene; and ITS: ribosomal internal transcribed spacer region of ribosomal DNA (ITS1-5.8S-ITS2).
[b] G: Gamma distributed rate variation among sites; GTR: Generalised time-reversible; HKY: Hasegawa-Kishino-Yano; I: Proportion of invariable sites.

Table S3. Sample size and haplotypes detected by distribution locality among *D. eres*.

Gene/Locus	BJ [a, b]	CQ	FJ	GS	HEB	HN	HUB	JS	JX	JL	LN	NX	SD	SC	YN	ZJ
EF1-α	11 (5)	2 (2)	3 (3)	2 (2)	2 (1)	6 (4)	33 (13)	6 (4)	16 (8)	11 (10)	2 (2)	2 (1)	6 (4)	6 (4)	3 (1)	21 (7)
CAL	11 (4)	2 (2)	3 (2)	2 (2)	2 (1)	5 (2)	39 (10)	6 (2)	10 (4)	10 (5)	2 (2)	2 (1)	6 (3)	6 (3)	3 (1)	9 (5)
TUB2	11 (6)	2 (2)	3 (3)	2 (2)	2 (1)	6 (4)	38 (8)	6 (5)	16 (9)	11 (2)	2 (2)	2 (2)	6 (5)	6 (3)	3 (1)	21 (3)
HIS	8 (3)	2 (2)	3 (2)	2 (2)	–	6 (4)	5 (3)	6 (4)	16 (4)	–	2 (2)	2 (1)	5 (5)	2 (1)	3 (1)	8 (7)
ITS	11 (7)	2 (2)	3 (3)	2 (2)	2 (2)	6 (5)	39 (14)	6 (5)	16 (14)	11 (4)	2 (2)	2 (1)	6 (6)	5 (4)	3 (1)	21 (12)

[a] BJ: Beijing; CQ: Chongqing; FJ: Fujian; GS: Gansu; HEB: Hebei; HN: Henan; HUB: Hubei; JS: Jiangsu; JX: Jiangxi; JL: Jilin; LN: Liaoning; NX: Ningxia; SD: Shandong; SC: Sichuan; YN: Yunnan; ZJ: Zhejiang.

[b] Number indicate sample size and numbers in parentheses indicate the haplotype diversity of each population.

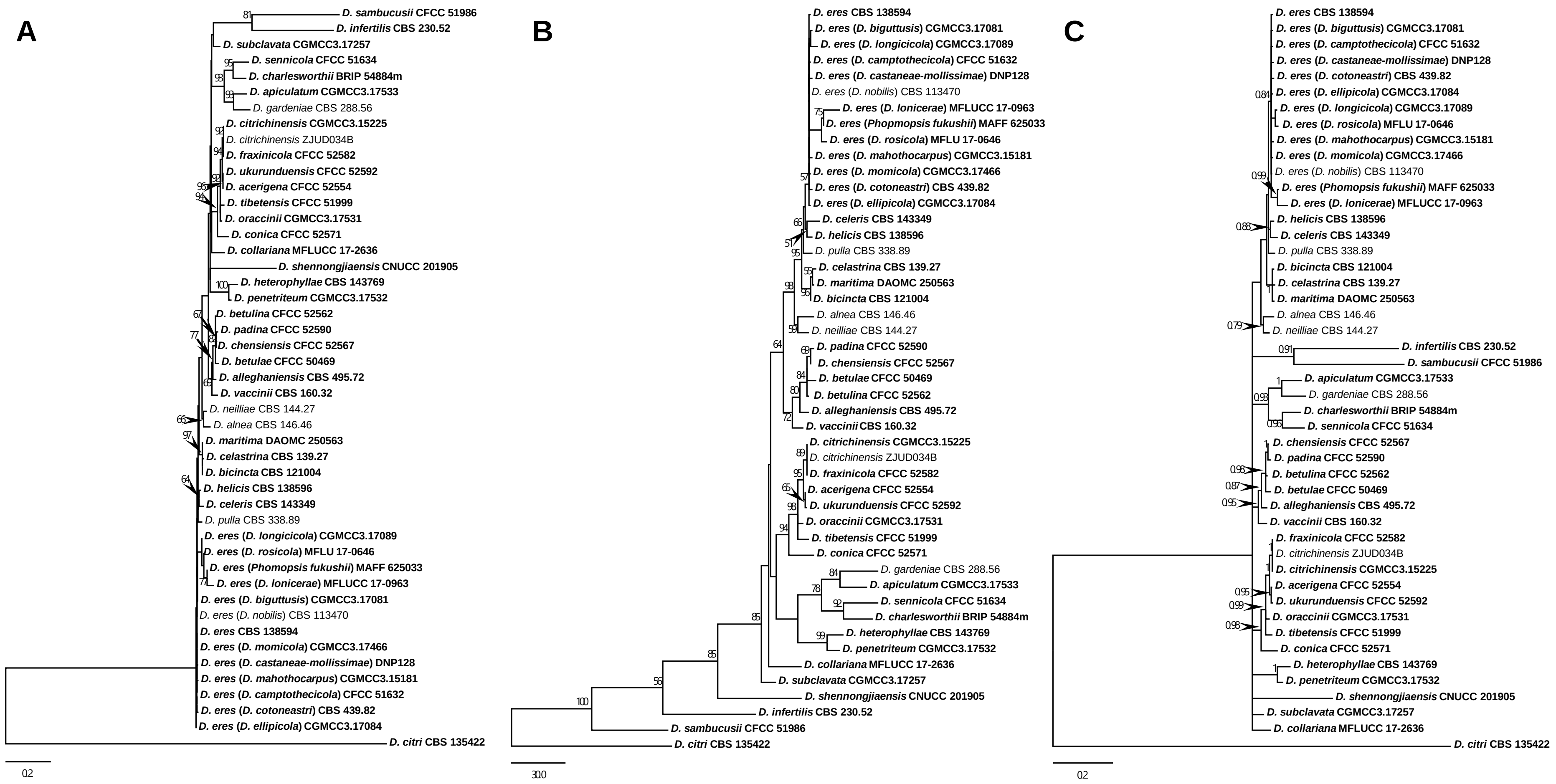

Figure S1. Phylogenetic analysis of the *D. eres* species complex based on the *EF1-α* locus. **A.** RAxML phylogenetic tree, **B.** Parsimonious phylogenetic tree, **C.** Bayesian phylogenetic tree. The trees were rooted using *D. citri* (CBS 135422). The Maximum likelihood and maximum parsimony bootstrap values (MLBS and MPBS)>50%, bayesian posterior probabilities values (BIPP)>0.75 are given at the branch nodes. Holotype, ex-type, ex-epitype, and ex-neotype cultures are indicated in isolate number with **bold** characters. The scale bar represents the expected number of changes per site.

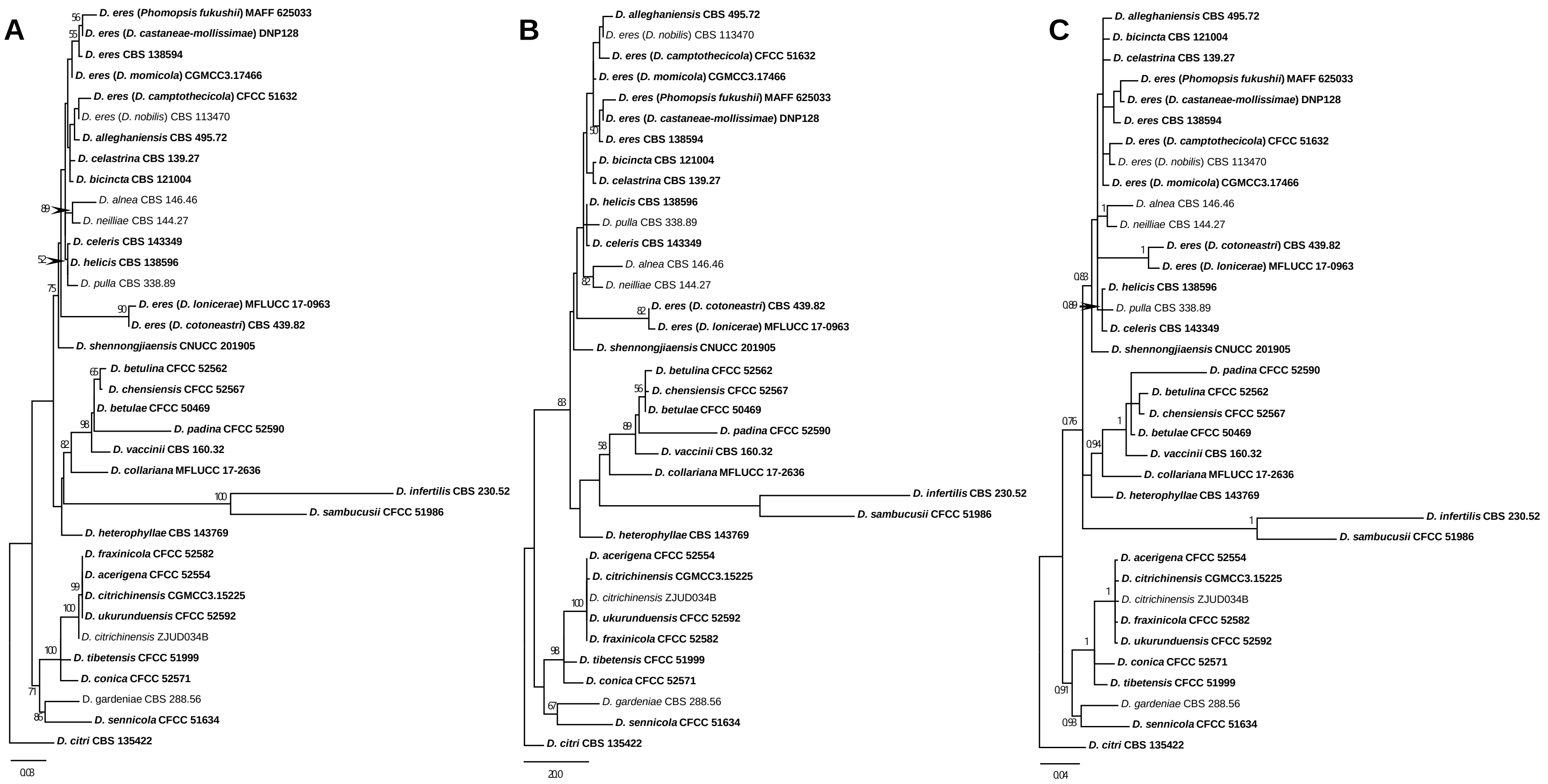

Figure S2. Phylogenetic analysis of the *D. eres* species complex based on the *CAL* locus. **A.** RAxML phylogenetic tree, **B.** Parsimonious phylogenetic tree, **C.** Bayesian phylogenetic tree. The trees were rooted using *D. citri* (CBS 135422). The Maximum likelihood and maximum parsimony bootstrap values (MLBS and MPBS)>50%, bayesian posterior probabilities values (BIPP)>0.75 are given at the branch nodes. Holotype, ex-type, ex-epitype, and ex-neotype cultures are indicated in isolate number with **bold** characters. The scale bar represents the expected number of changes per site.

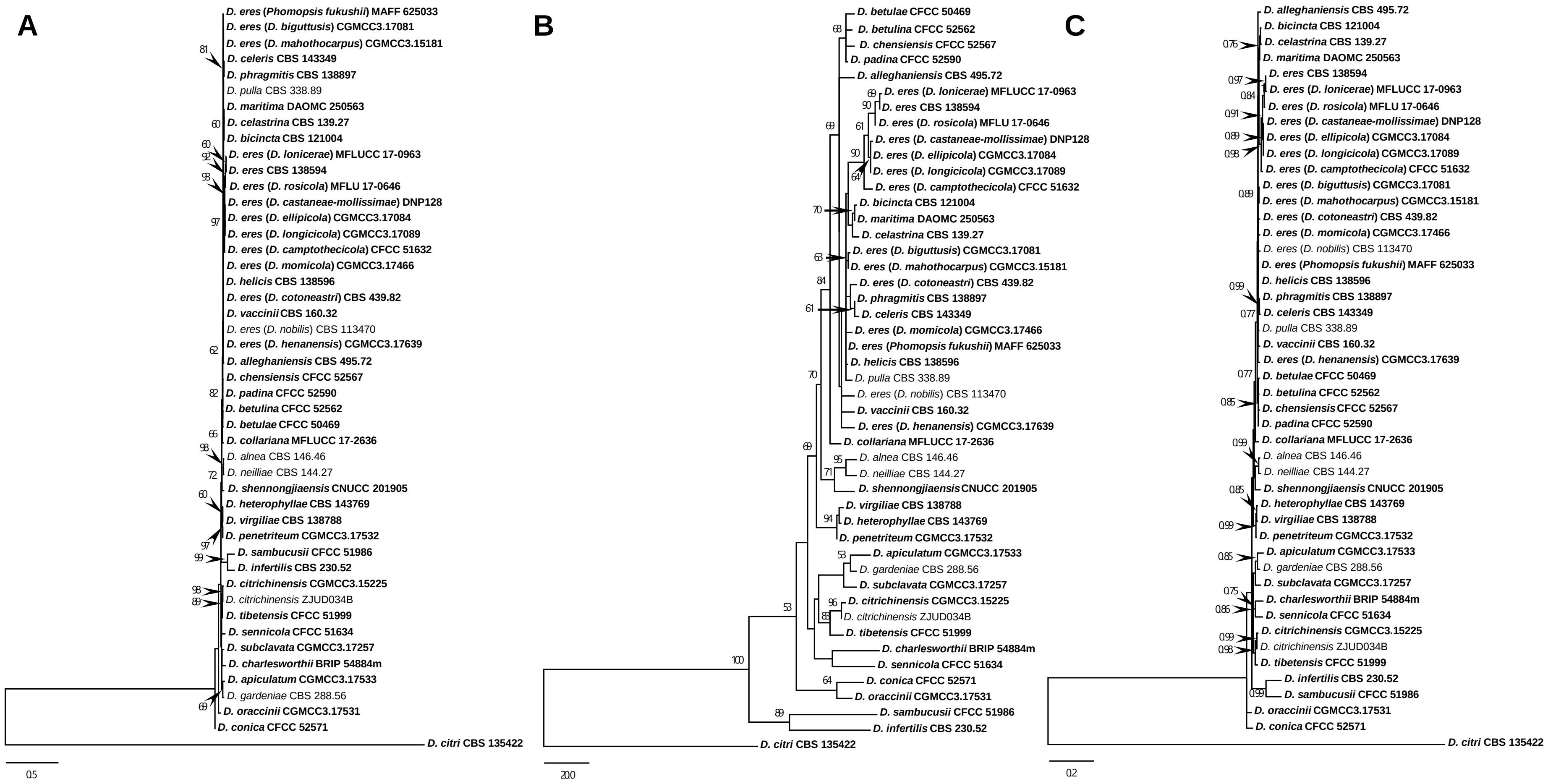

Figure S3. Phylogenetic analysis of the *D. eres* species complex based on the *TUB2* locus. **A.** RAxML phylogenetic tree, **B.** Parsimonious phylogenetic tree, **C.** Bayesian phylogenetic tree. The trees were rooted using *D. citri* (CBS 135422). The Maximum likelihood and maximum parsimony bootstrap values (MLBS and MPBS)>50%, bayesian posterior probabilities values (BIPP)>0.75 are given at the branch nodes. Holotype, ex-type, ex-epitype, and ex-neotype cultures are indicated in isolate number with **bold** characters. The scale bar represents the expected number of changes per site.

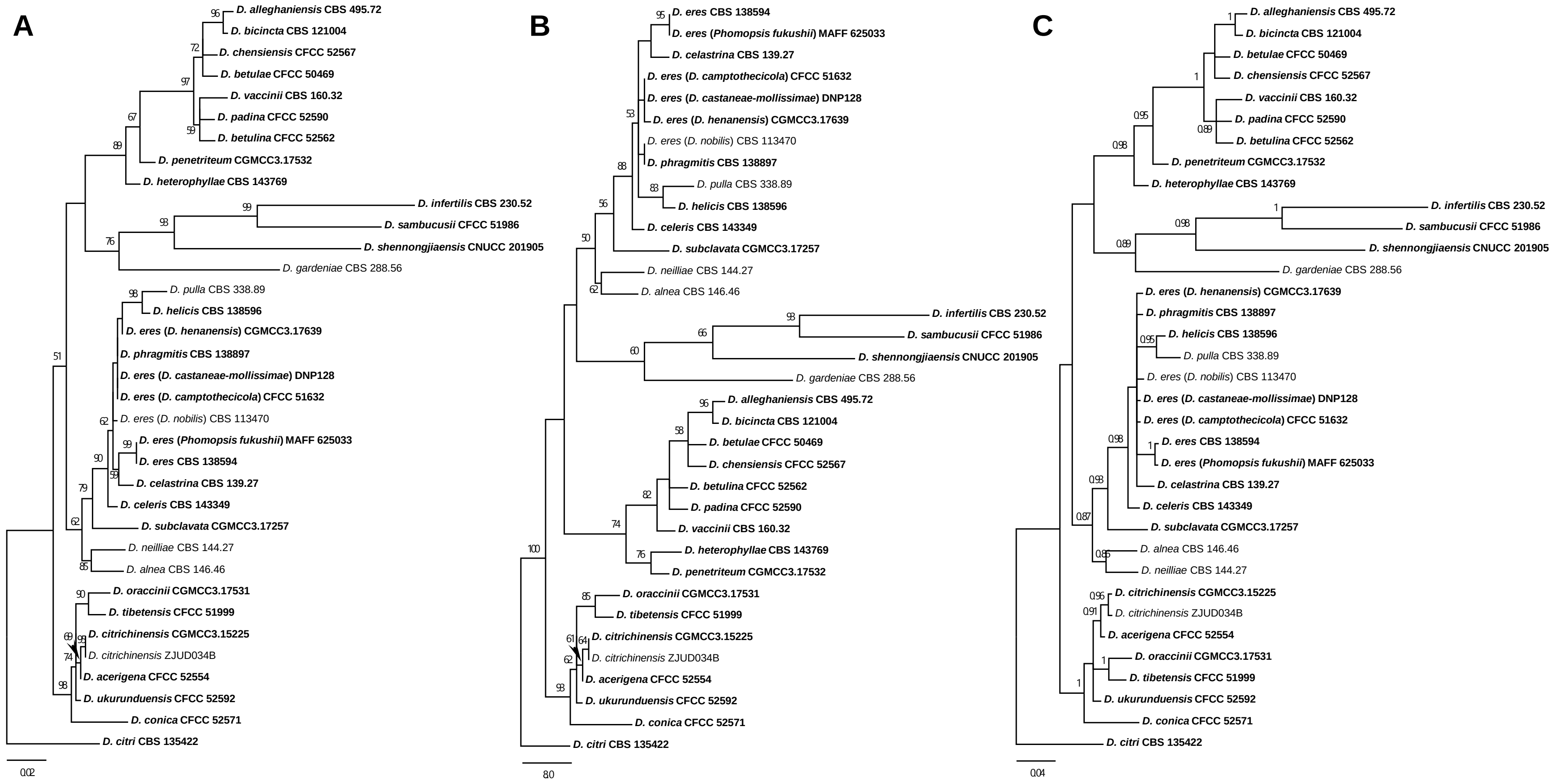

Figure S4. Phylogenetic analysis of the *D. eres* species complex based on the *HIS* locus. **A.** RAxML phylogenetic tree, **B.** Parsimonious phylogenetic tree, **C.** Bayesian phylogenetic tree. The trees were rooted using *D. citri* (CBS 135422). The Maximum likelihood and maximum parsimony bootstrap values (MLBS and MPBS)>50%, bayesian posterior probabilities values (BIPP)>0.75 are given at the branch nodes. Holotype, ex-type, ex-epitype, and ex-neotype cultures are indicated in isolate number with **bold** characters. The scale bar represents the expected number of changes per site.

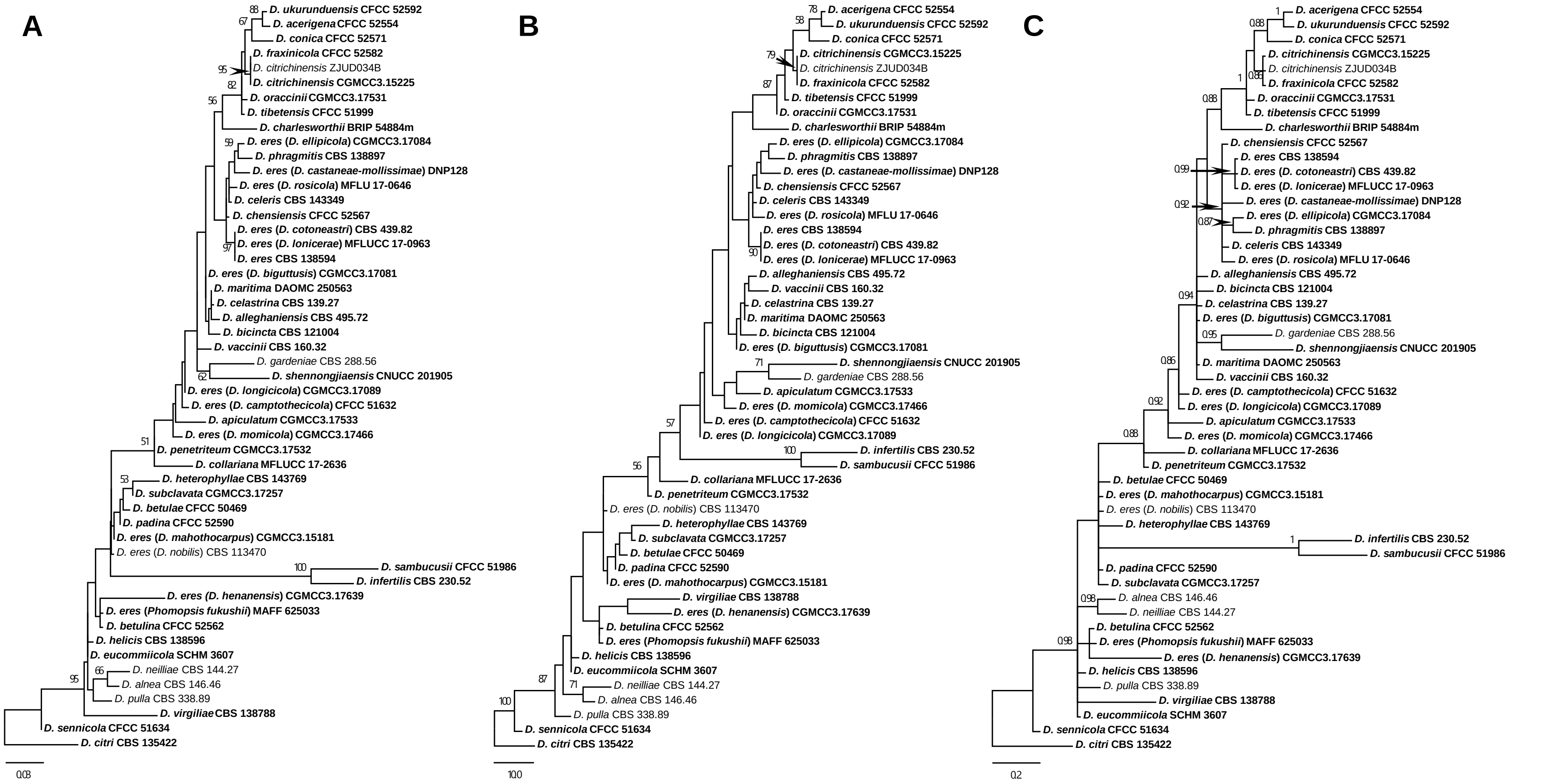

Figure S5. Phylogenetic analysis of the *D. eres* species complex based on the ITS locus. **A.** RAxML phylogenetic tree, **B.** Parsimonious phylogenetic tree, **C.** Bayesian phylogenetic tree. The trees were rooted using *D. citri* (CBS 135422). The Maximum likelihood and maximum parsimony bootstrap values (MLBS and MPBS)>50%, bayesian posterior probabilities values (BIPP)>0.75 are given at the branch nodes. Holotype, ex-type, ex-epitype, and ex-neotype cultures are indicated in isolate number with **bold** characters. The scale bar represents the expected number of changes per site.

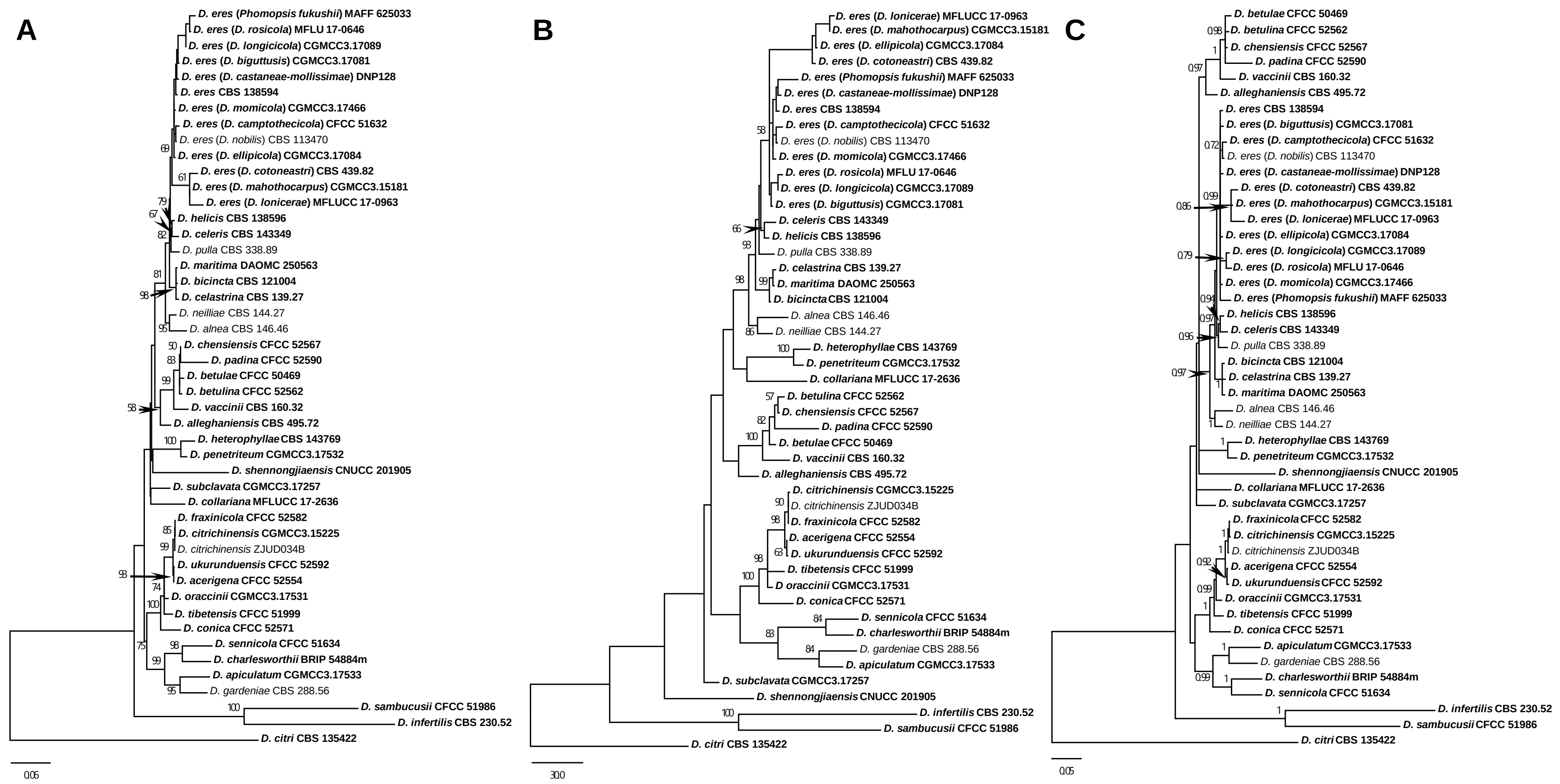

Figure S6. Phylogenetic analysis of the *D. eres* species complex based on the *EF1-α+CAL* loci. **A.** RAxML phylogenetic tree, **B.** Parsimonious phylogenetic tree, **C.** Bayesian phylogenetic tree. The trees were rooted using *D. citri* (CBS 135422). The Maximum likelihood and maximum parsimony bootstrap values (MLBS and MPBS)>50%, bayesian posterior probabilities values (BIPP)>0.75 are given at the branch nodes. Holotype, ex-type, ex-epitype, and ex-neotype cultures are indicated in isolate number with **bold** characters. The scale bar represents the expected number of changes per site.

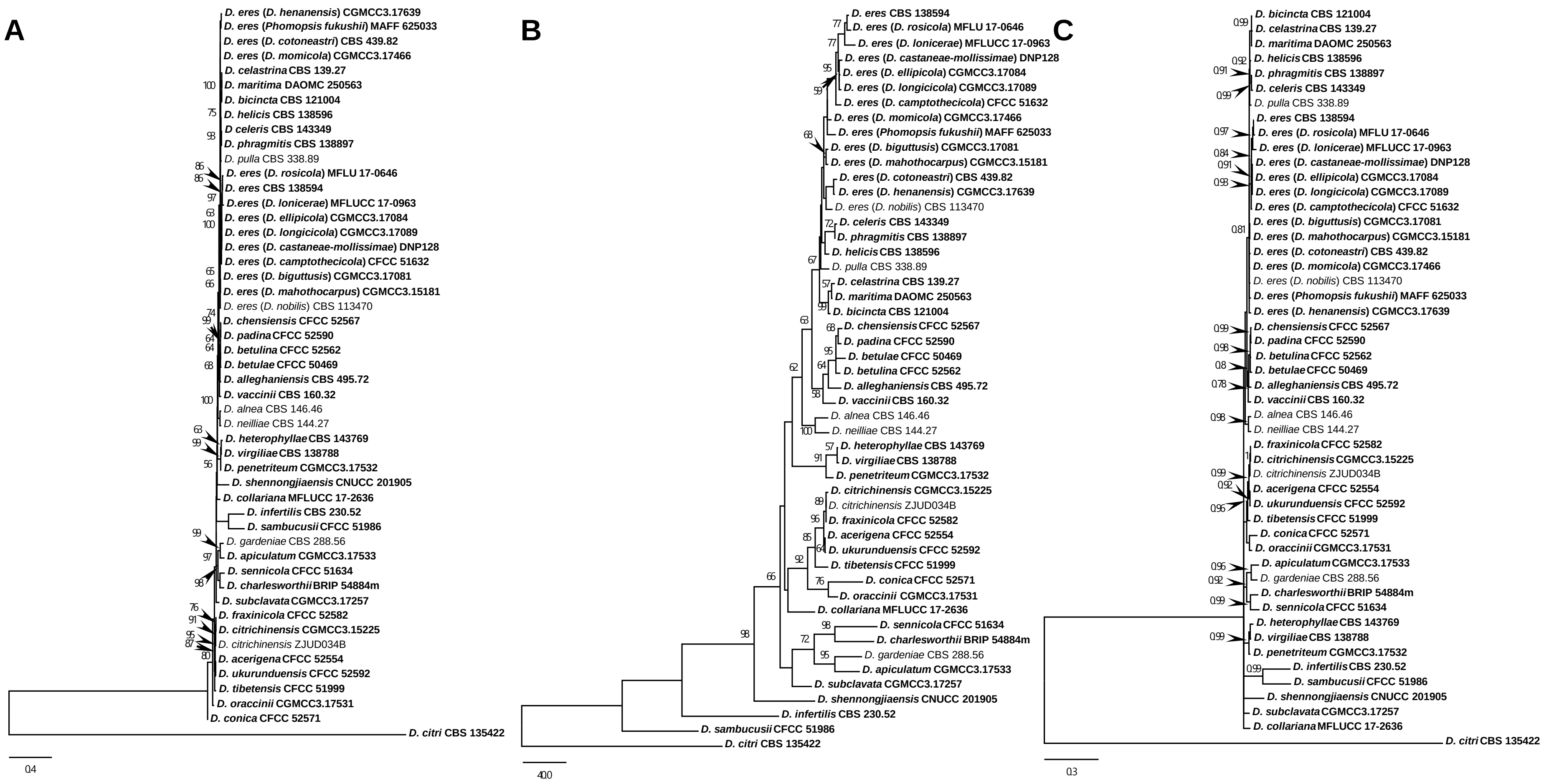

Figure S7. Phylogenetic analysis of the *D. eres* species complex based on the *EF1-α+TUB2* loci. **A.** RAxML phylogenetic tree, **B.** Parsimonious phylogenetic tree, **C.** Bayesian phylogenetic tree. The trees were rooted using *D. citri* (CBS 135422). The Maximum likelihood and maximum parsimony bootstrap values (MLBS and MPBS)>50%, bayesian posterior probabilities values (BIPP)>0.75 are given at the branch nodes. Holotype, ex-type, ex-epitype, and ex-neotype cultures are indicated in isolate number with **bold** characters. The scale bar represents the expected number of changes per site.

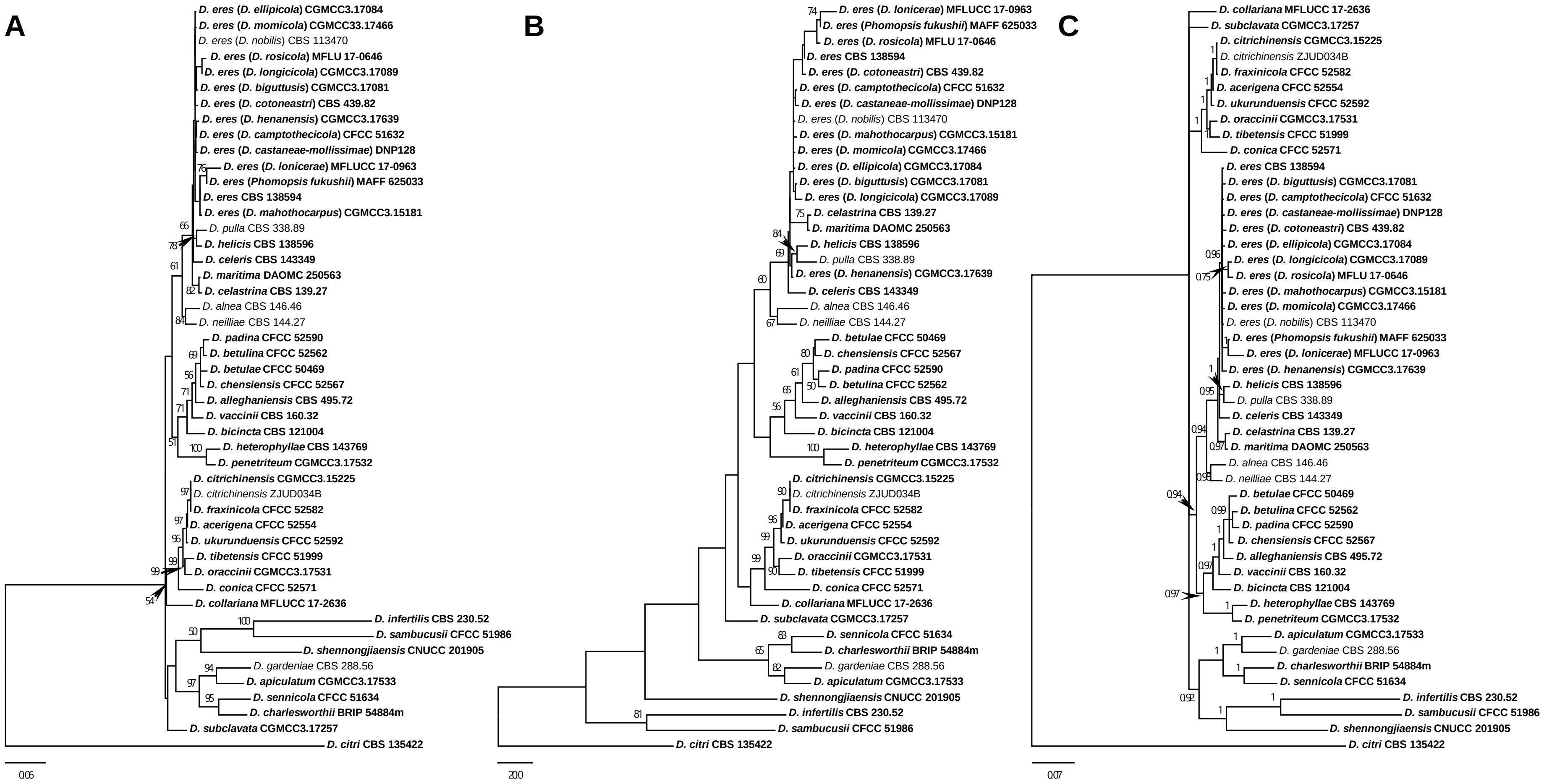

Figure S8. Phylogenetic analysis of the *D. eres* species complex based on the *EF1-α+HIS* loci. **A.** RAxML phylogenetic tree, **B.** Parsimonious phylogenetic tree, **C.** Bayesian phylogenetic tree. The trees were rooted using *D. citri* (CBS 135422). The Maximum likelihood and maximum parsimony bootstrap values (MLBS and MPBS)>50%, bayesian posterior probabilities values (BIPP)>0.75 are given at the branch nodes. Holotype, ex-type, ex-epitype, and ex-neotype cultures are indicated in isolate number with **bold** characters. The scale bar represents the expected number of changes per site.

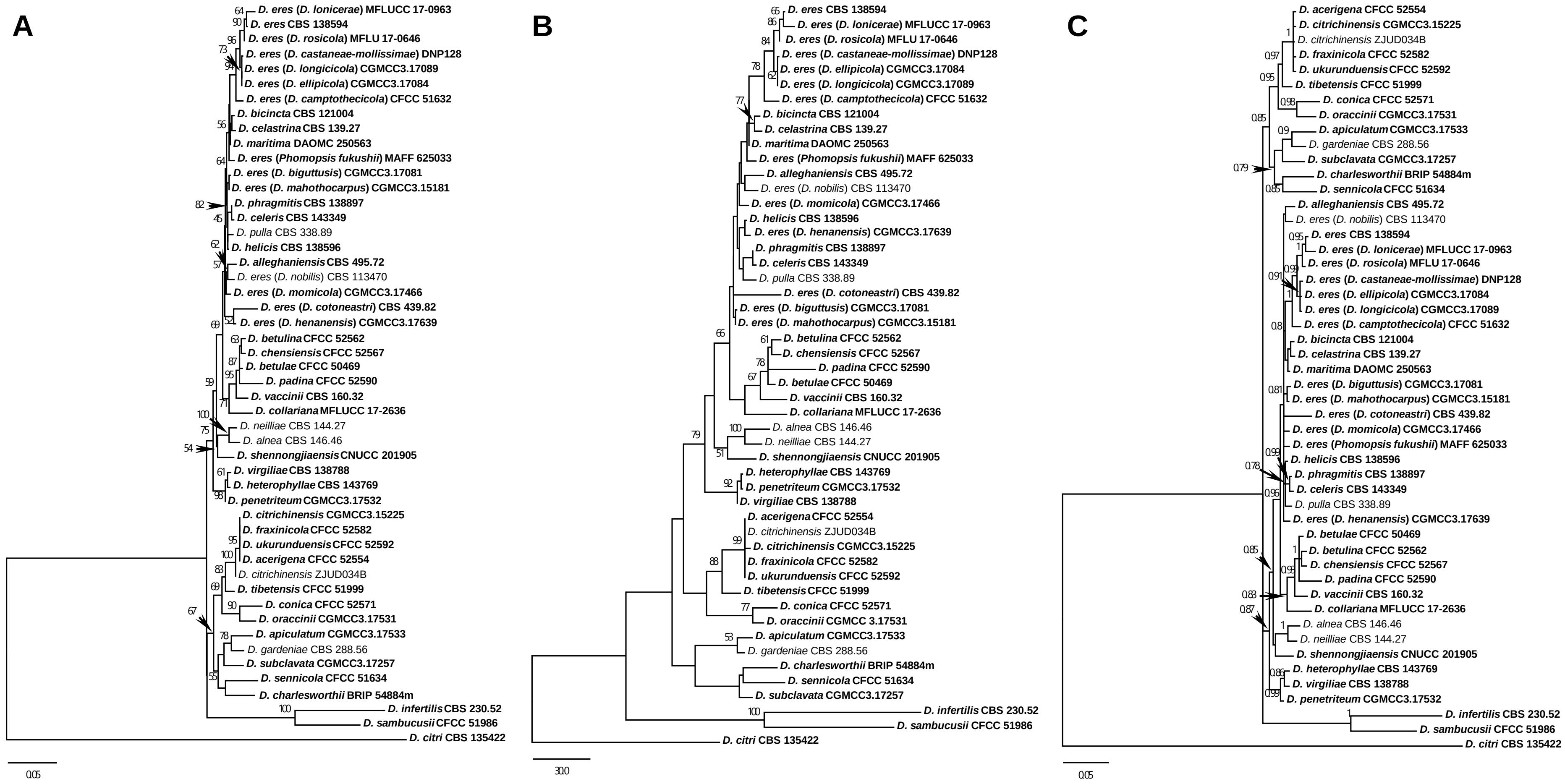

Figure S9. Phylogenetic analysis of the *D. eres* species complex based on the *CAL+TUB2* loci. **A.** RAxML phylogenetic tree, **B.** Parsimonious phylogenetic tree, **C.** Bayesian phylogenetic tree. The trees were rooted using *D. citri* (CBS 135422). The Maximum likelihood and maximum parsimony bootstrap values (MLBS and MPBS)>50%, bayesian posterior probabilities values (BIPP)>0.75 are given at the branch nodes. Holotype, ex-type, ex-epitype, and ex-neotype cultures are indicated in isolate number with **bold** characters. The scale bar represents the expected number of changes per site.

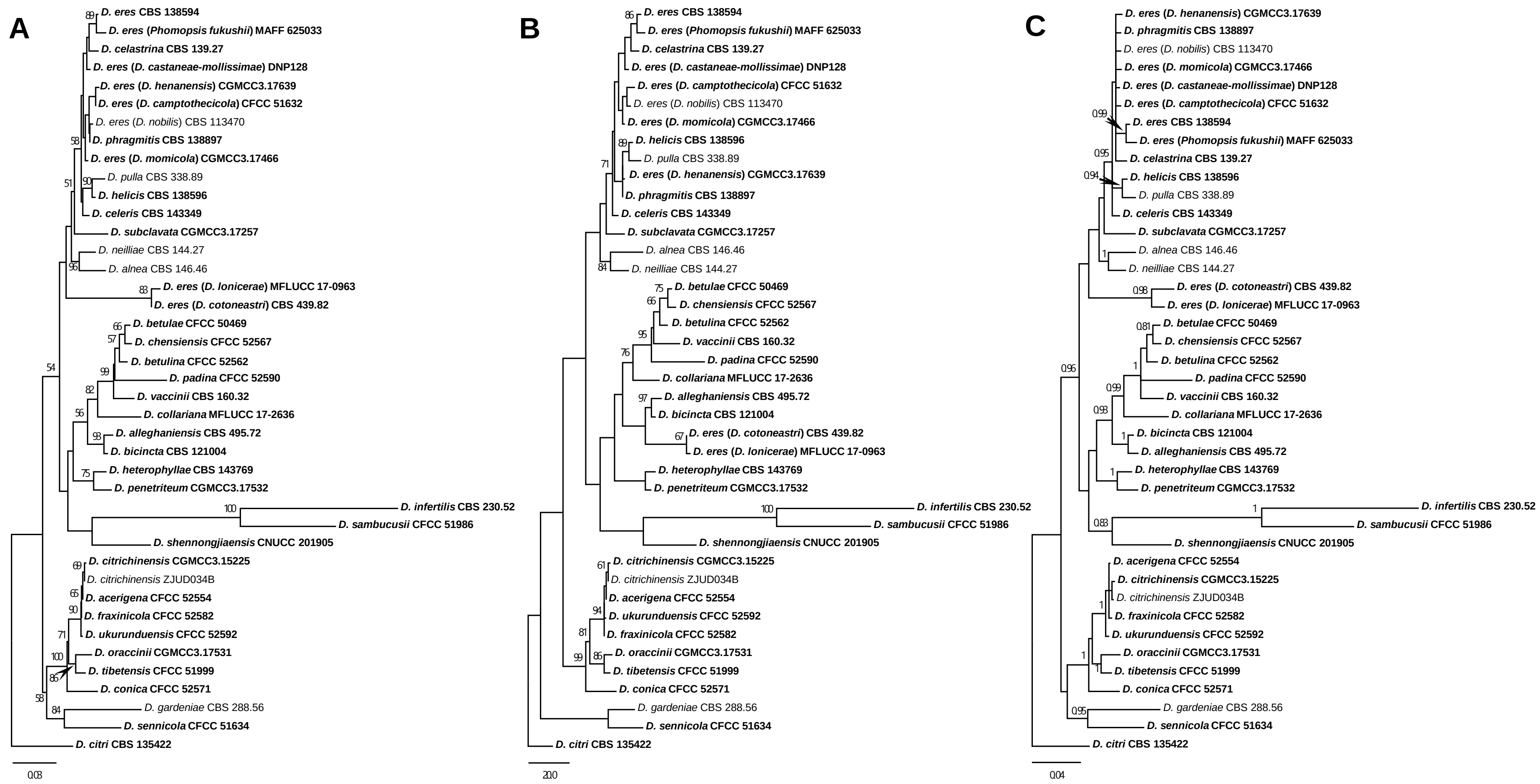

Figure S10. Phylogenetic analysis of the *D. eres* species complex based on the *CAL+HIS* loci. **A.** RAxML phylogenetic tree, **B.** Parsimonious phylogenetic tree, **C.** Bayesian phylogenetic tree. The trees were rooted using *D. citri* (CBS 135422). The Maximum likelihood and maximum parsimony bootstrap values (MLBS and MPBS)>50%, bayesian posterior probabilities values (BIPP)>0.75 are given at the branch nodes. Holotype, ex-type, ex-epitype, and ex-neotype cultures are indicated in isolate number with **bold** characters. The scale bar represents the expected number of changes per site.

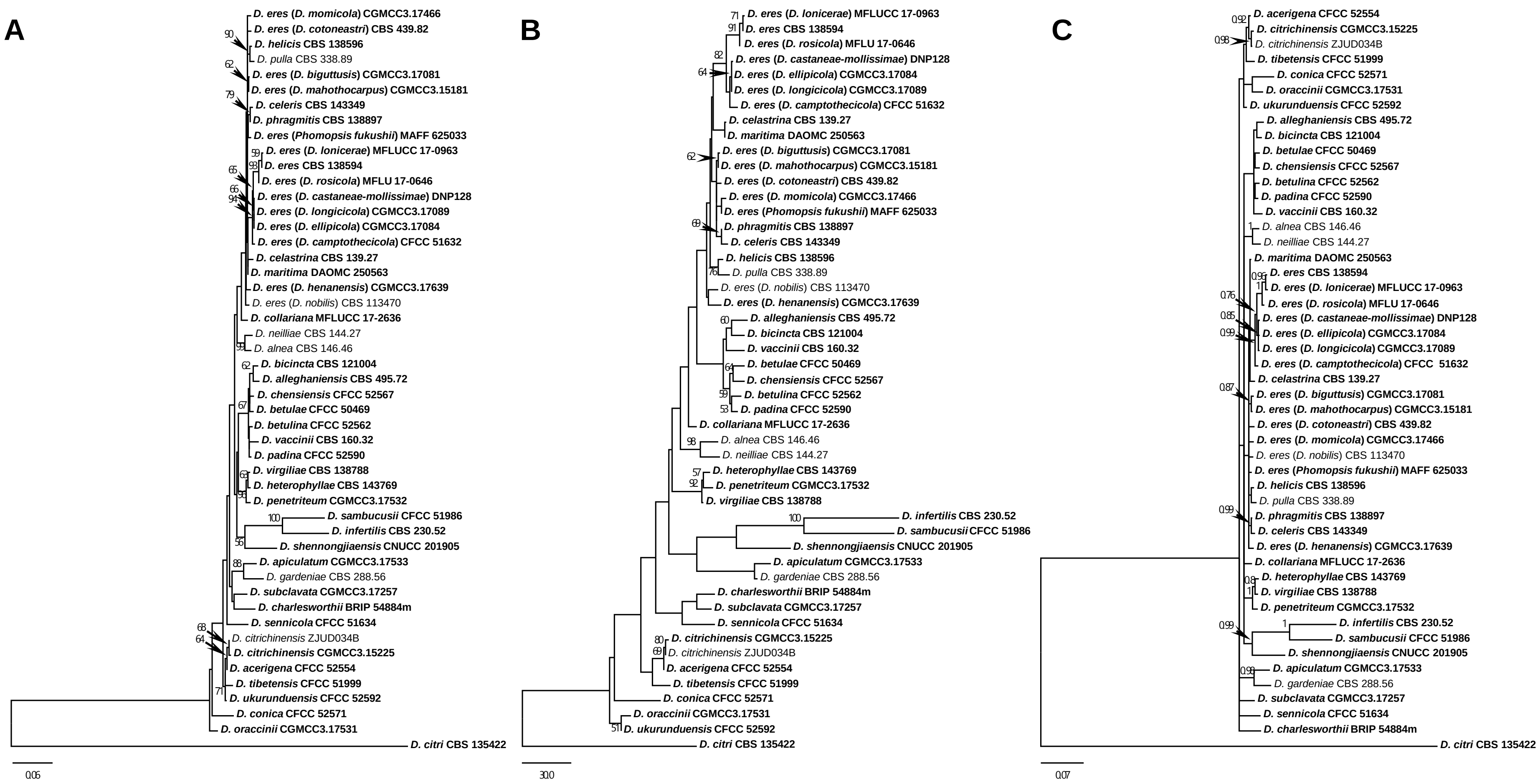

Figure S11. Phylogenetic analysis of the *D. eres* species complex based on the *TUB2+HIS* loci. **A.** RAxML phylogenetic tree, **B.** Parsimonious phylogenetic tree, **C.** Bayesian phylogenetic tree. The trees were rooted using *D. citri* (CBS 135422). The Maximum likelihood and maximum parsimony bootstrap values (MLBS and MPBS)>50%, bayesian posterior probabilities values (BIPP)>0.75 are given at the branch nodes. Holotype, ex-type, ex-epitype, and ex-neotype cultures are indicated in isolate number with **bold** characters. The scale bar represents the expected number of changes per site.

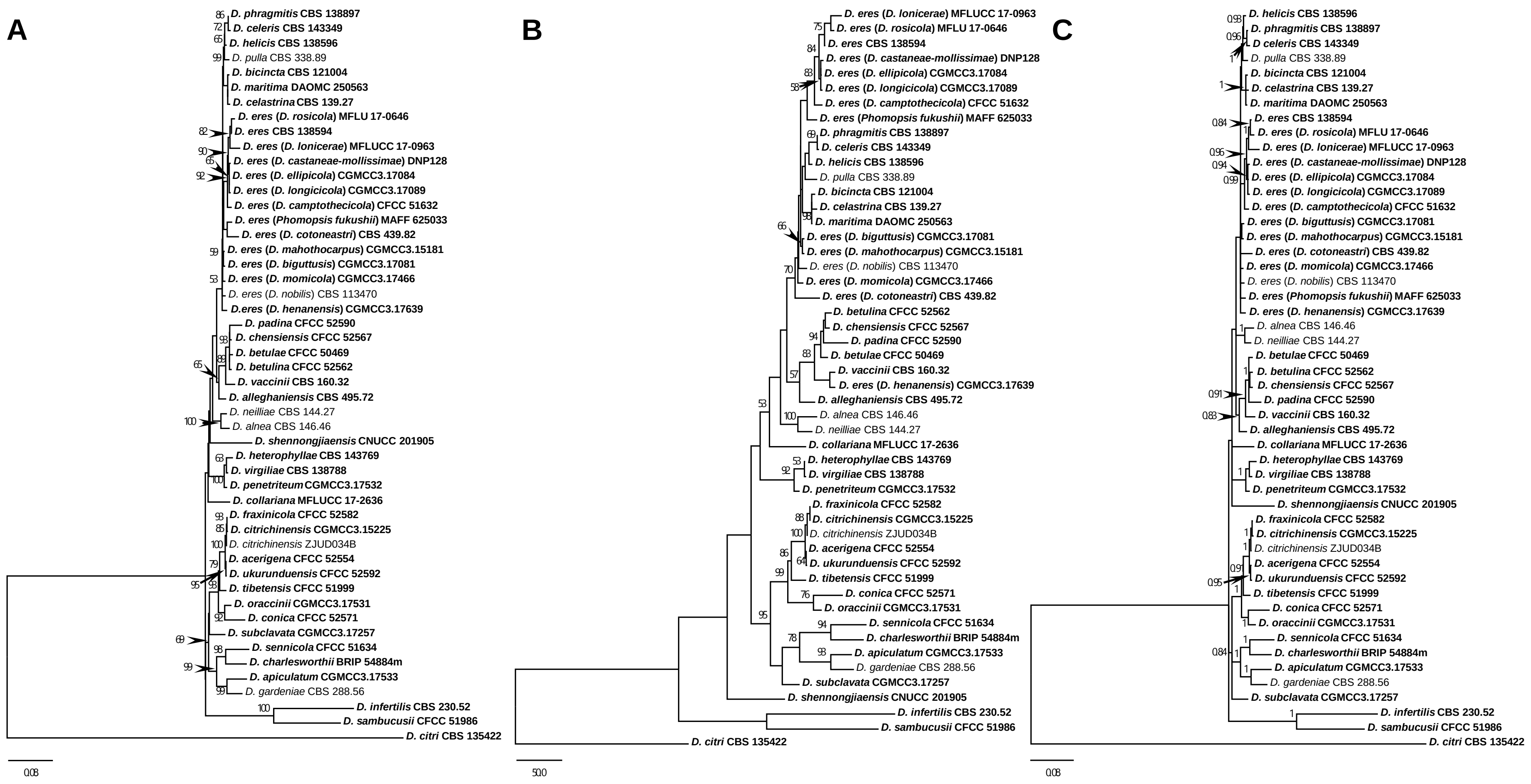

Figure S12. Phylogenetic analysis of the *D. eres* species complex based on the *EF1-α+CAL+TUB2* loci. **A.** RAxML phylogenetic tree, **B.** Parsimonious phylogenetic tree, **C.** Bayesian phylogenetic tree. The trees were rooted using *D. citri* (CBS 135422). The Maximum likelihood and maximum parsimony bootstrap values (MLBS and MPBS)>50%, bayesian posterior probabilities values (BIPP)>0.75 are given at the branch nodes. Holotype, ex-type, ex-epitype, and ex-neotype cultures are indicated in isolate number with **bold** characters. The scale bar represents the expected number of changes per site.

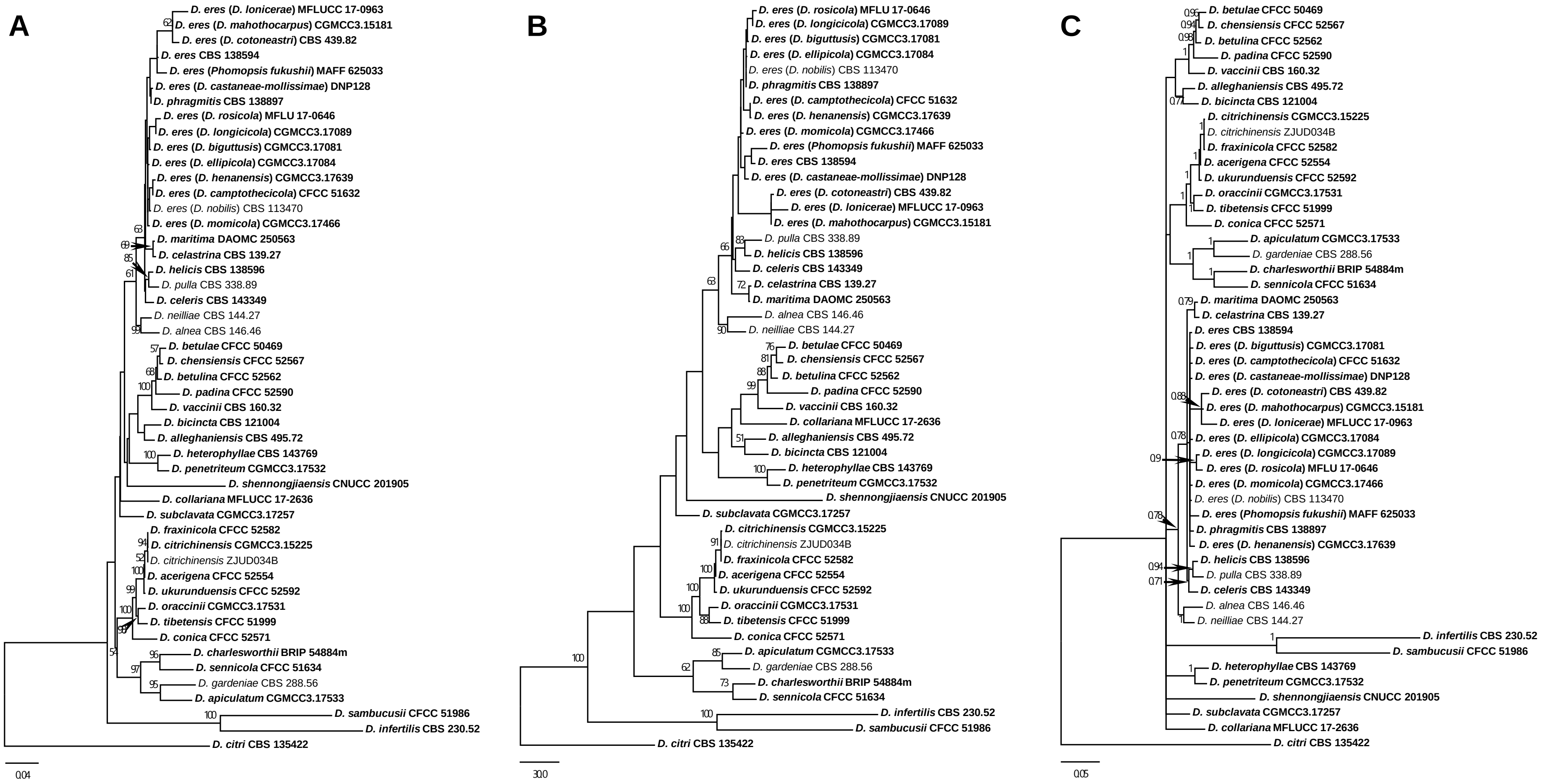

Figure S13. Phylogenetic analysis of the *D. eres* species complex based on the *EF1-α+CAL+HIS* loci. **A.** RAxML phylogenetic tree, **B.** Parsimonious phylogenetic tree, **C.** Bayesian phylogenetic tree. The trees were rooted using *D. citri* (CBS 135422). The Maximum likelihood and maximum parsimony bootstrap values (MLBS and MPBS)>50%, bayesian posterior probabilities values (BIPP)>0.75 are given at the branch nodes. Holotype, ex-type, ex-epitype, and ex-neotype cultures are indicated in isolate number with **bold** characters. The scale bar represents the expected number of changes per site.

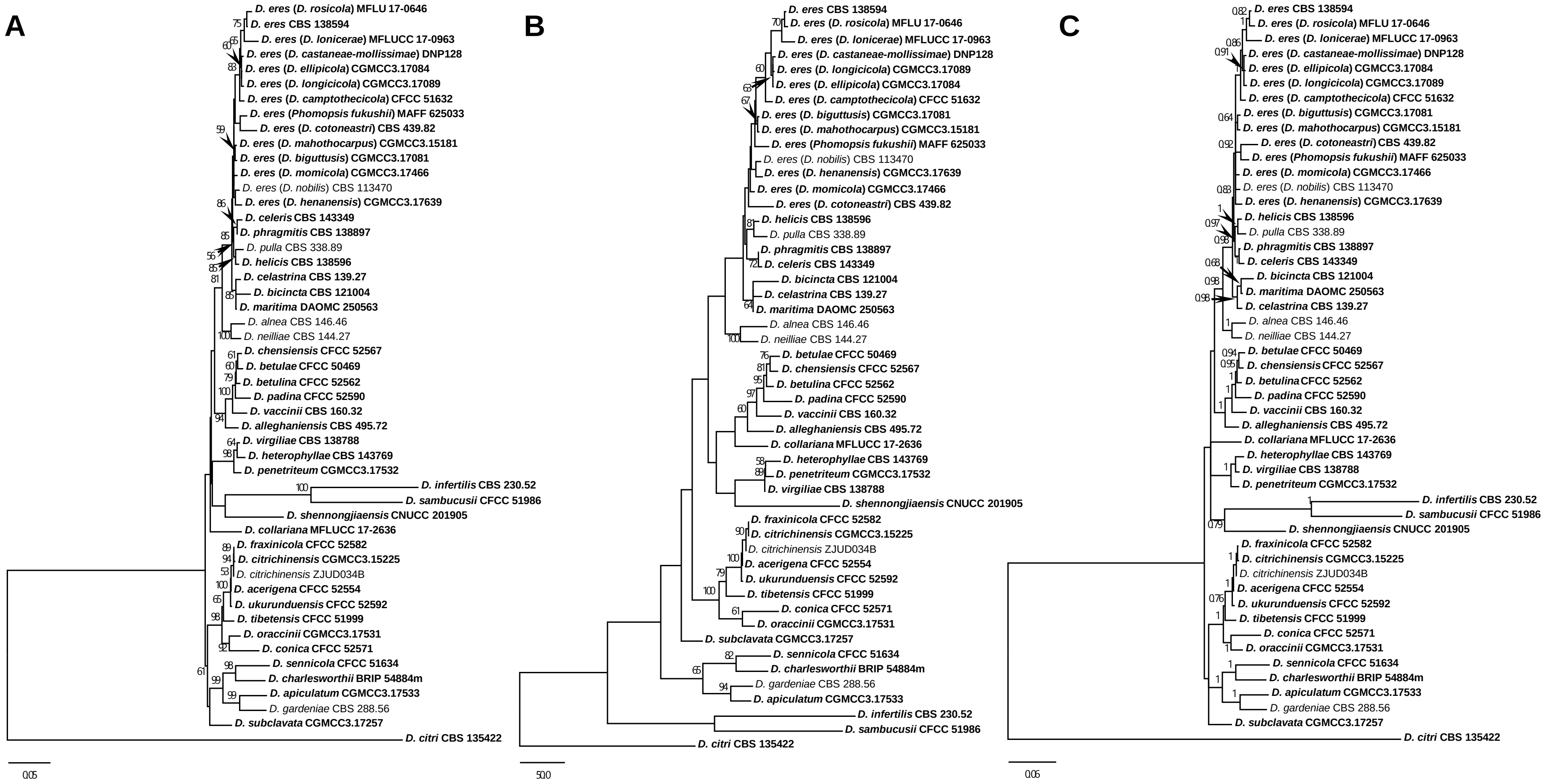

Figure S14. Phylogenetic analysis of the *D. eres* species complex based on the *EF1-α+CAL+TUB2+HIS* loci. **A.** RAxML phylogenetic tree, **B.** Parsimonious phylogenetic tree, **C.** Bayesian phylogenetic tree. The trees were rooted using *D. citri* (CBS 135422). The Maximum likelihood and maximum parsimony bootstrap values (MLBS and MPBS)>50%, bayesian posterior probabilities values (BIPP)>0.75 are given at the branch nodes. Holotype, ex-type, ex-epitype, and ex-neotype cultures are indicated in isolate number with **bold** characters. The scale bar represents the expected number of changes per site.

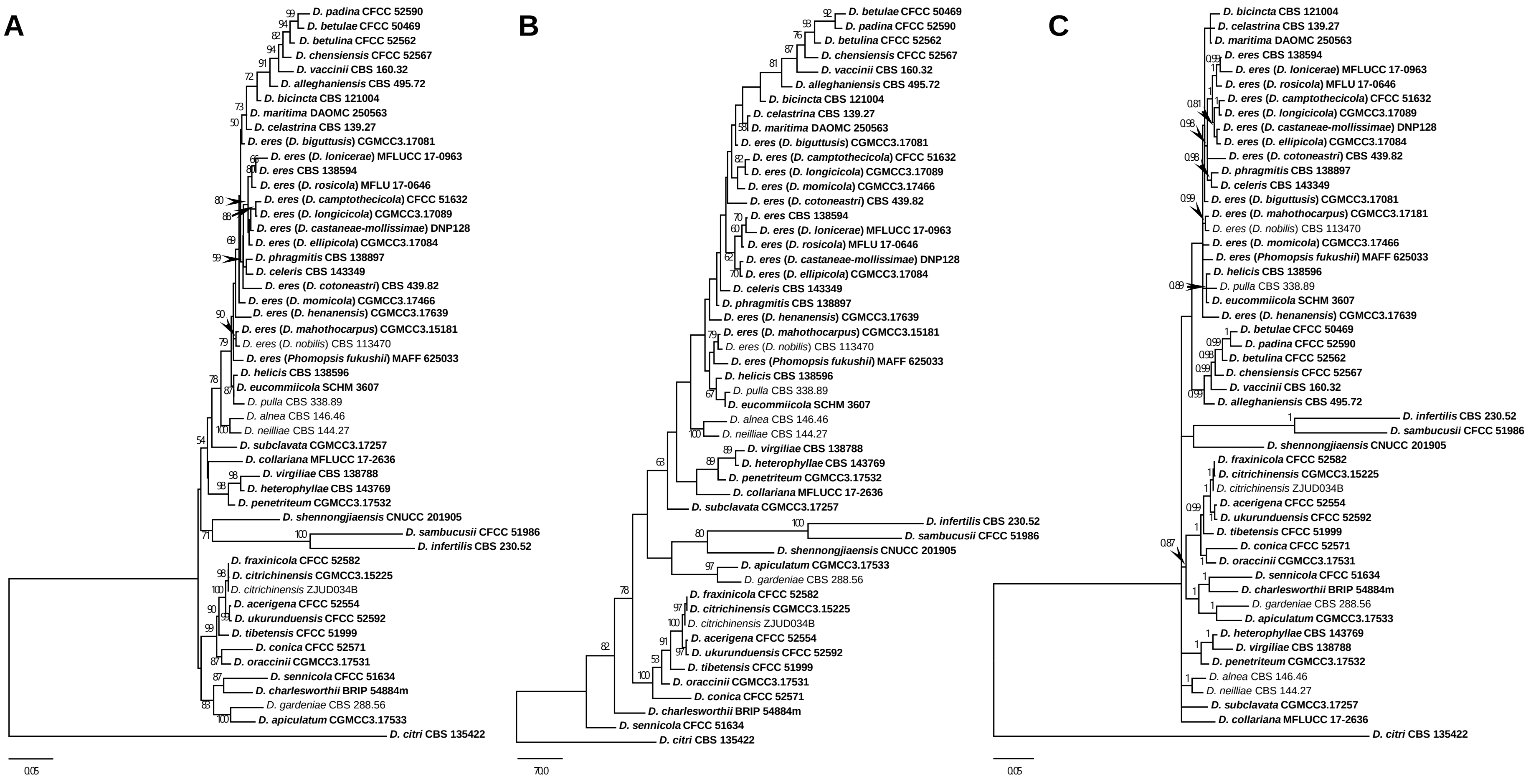

Figure S15. Phylogenetic analysis of the *D. eres* species complex based on the *EF1-α*+*CAL*+*TUB2*+*HIS*+ITS loci. **A.** RAxML phylogenetic tree, **B.** Parsimonious phylogenetic tree, **C.** Bayesian phylogenetic tree. The trees were rooted using *D. citri* (CBS 135422). The Maximum likelihood and maximum parsimony bootstrap values (MLBS and MPBS)>50%, bayesian posterior probabilities values (BIPP)>0.75 are given at the branch nodes. Holotype, ex-type, ex-epitype, and ex-neotype cultures are indicated in isolate number with **bold** characters. The scale bar represents the expected number of changes per site.

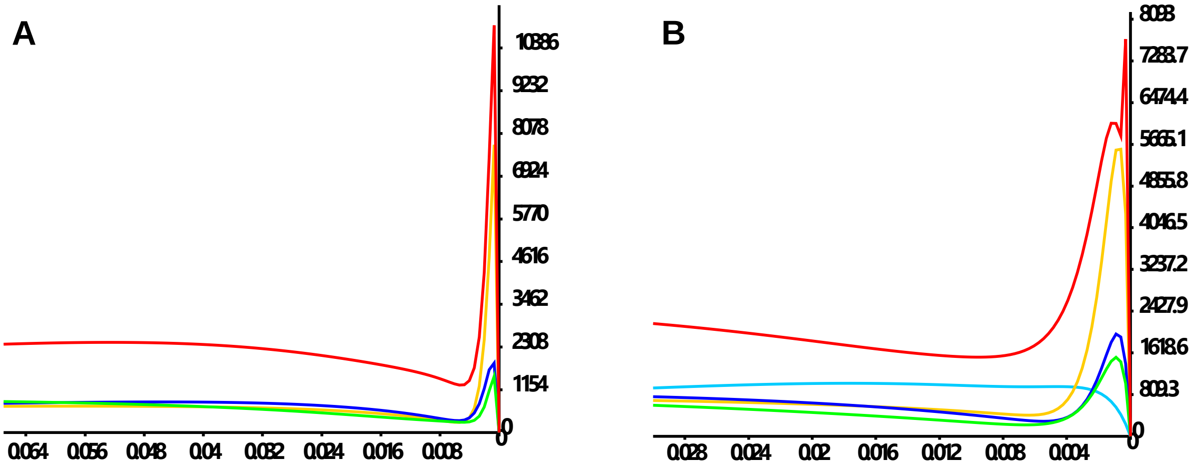

Figure S16. Phylogenetic informativeness profiles of 23 *Diaporthe* species including *D. eres* species complex and close species. The phylogenetic informativeness was shown as phylogenetic informativeness per site (PI per site) based on combined multi-locus dataset. **A.** Combined dataset from four loci *EF1-α*+*CAL*+*TUB2*+*HIS*. **B.** Combined dataset from five loci *EF1-α*+*CAL*+*TUB2*+*HIS*+ITS. Values on the X-axes correspond to the relative timescale (0-1). Values on the Y-axes represent 10^{-3} PI per site in arbitrary units.

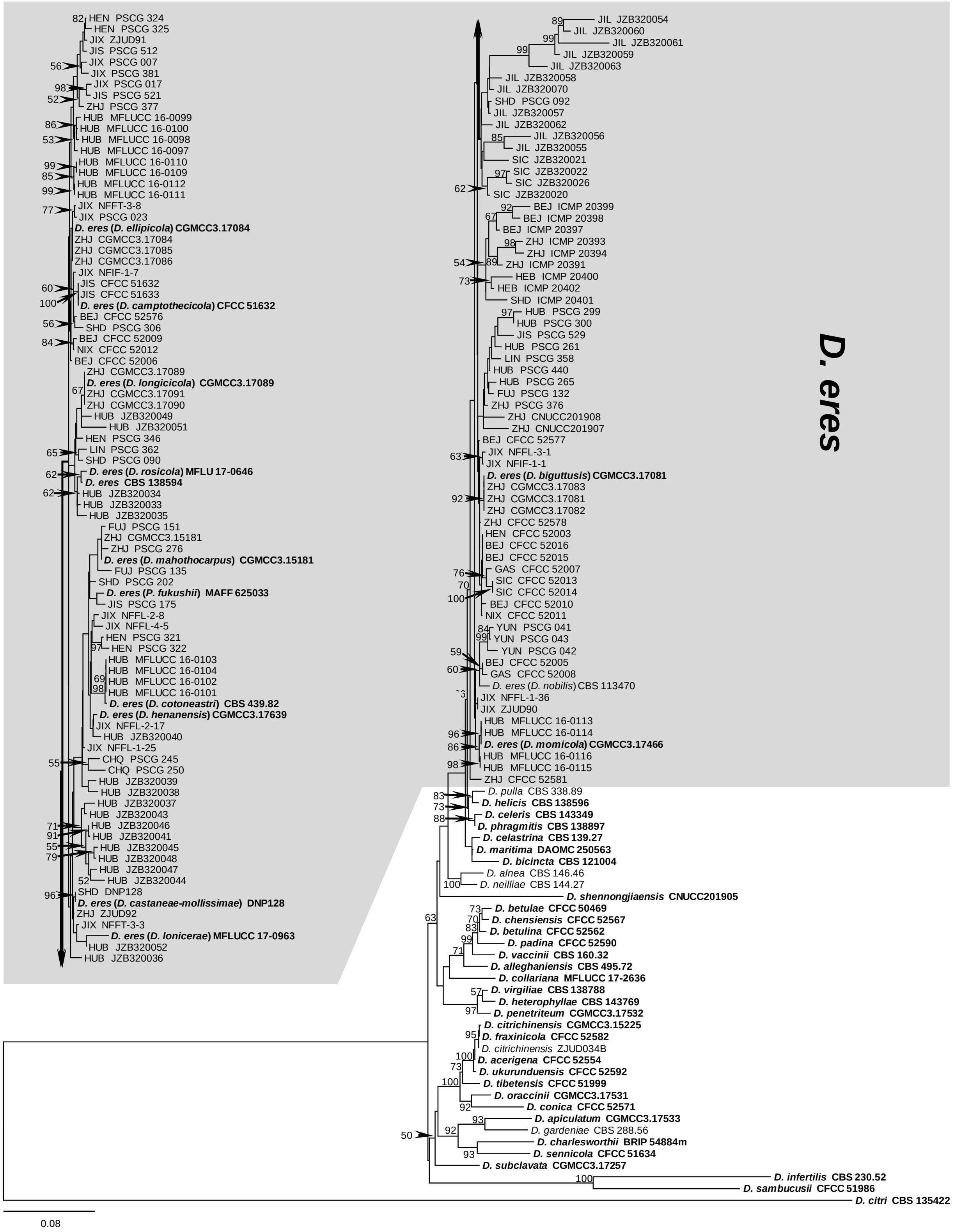

Figure S17. Phylogenetic tree generated from maximum likelihood analysis of all available type species of *Diaporthe* 51 isolates (Table 1) together with 138 isolates (Table S1), based on combined sequences of *EF1-α*, *CAL*, *TUB2*, and *HIS*.

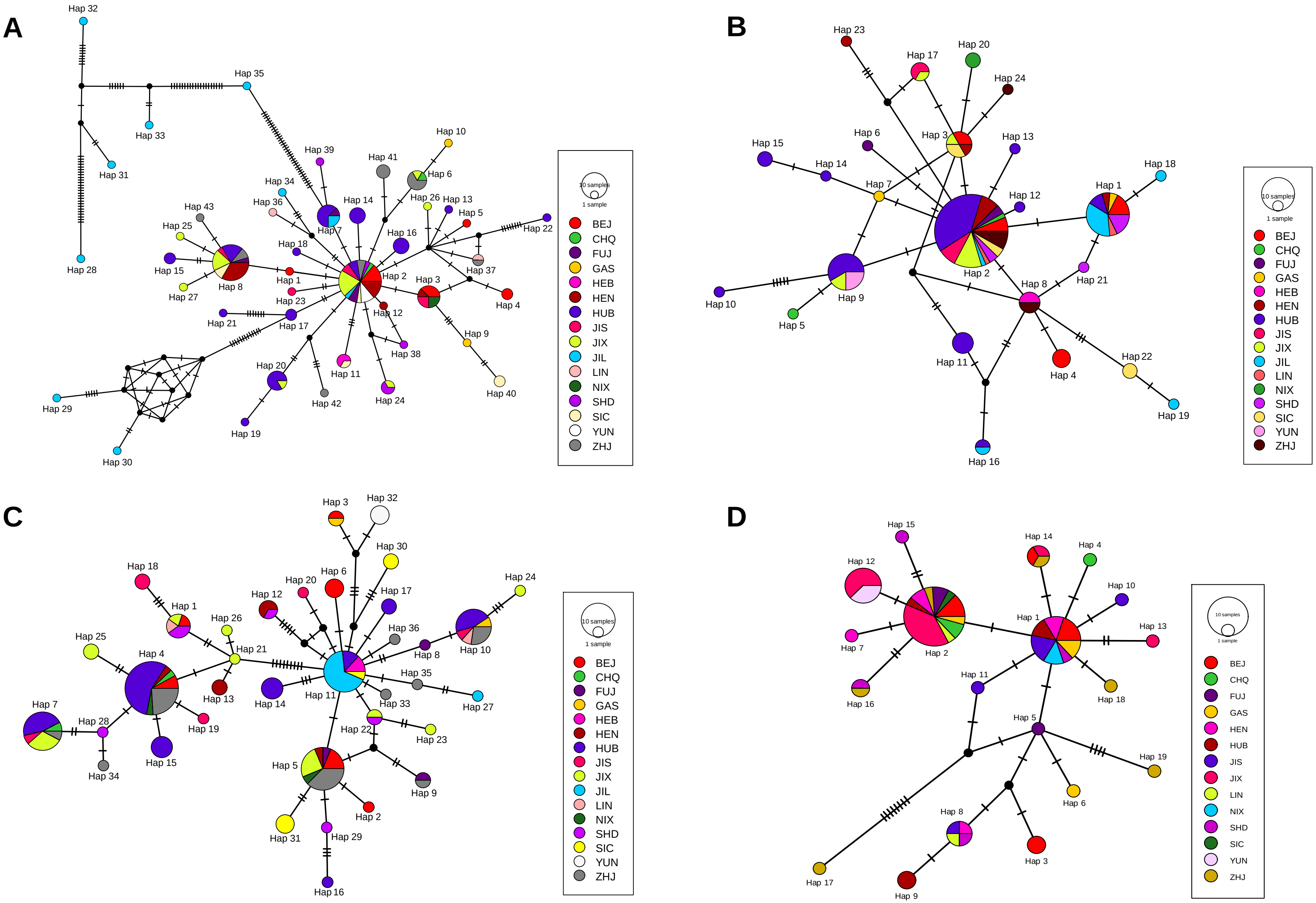
A
Hap 32
Hap 35
Hap 33
Hap 31
Hap 28
Hap 39
Hap 41
Hap 10
Hap 6
Hap 34
Hap 36
Hap 14
Hap 26
Hap 13
Hap 5
Hap 22
Hap 43
Hap 7
Hap 16
Hap 25
Hap 18
Hap 37
Hap 15
Hap 8
Hap 1
Hap 2
Hap 3
Hap 4
Hap 27
Hap 23
Hap 12
Hap 21
Hap 17
Hap 9
Hap 38
Hap 20
Hap 11
Hap 40
Hap 29
Hap 42
Hap 24
Hap 19
Hap 30
10 samples
1 sample
BEJ
CHQ
FUJ
GAS
HEB
HEN
HUB
JIS
JIX
JIL
LIN
NIX
SHD
SIC
YUN
ZHJ
B
Hap 23
Hap 17
Hap 20
Hap 24
Hap 15
Hap 6
Hap 3
Hap 13
Hap 14
Hap 7
Hap 12
Hap 18
Hap 1
Hap 2
Hap 21
Hap 8
Hap 9
Hap 10
Hap 5
Hap 11
Hap 4
Hap 22
Hap 19
Hap 16
10 samples
1 sample
BEJ
CHQ
FUJ
GAS
HEB
HEN
HUB
JIS
JIX
JIL
LIN
NIX
SHD
SIC
YUN
ZHJ
C
Hap 3
Hap 32
Hap 30
Hap 18
Hap 6
Hap 20
Hap 17
Hap 24
Hap 1
Hap 12
Hap 26
Hap 36
Hap 25
Hap 8
Hap 10
Hap 21
Hap 4
Hap 13
Hap 14
Hap 11
Hap 35
Hap 7
Hap 33
Hap 27
Hap 28
Hap 19
Hap 22
Hap 23
Hap 15
Hap 34
Hap 5
Hap 9
Hap 2
Hap 31
Hap 29
Hap 16
10 samples
1 sample
BEJ
CHQ
FUJ
GAS
HEB
HEN
HUB
JIS
JIX
JIL
LIN
NIX
SHD
SIC
YUN
ZHJ
D
Hap 15
Hap 14
Hap 4
Hap 12
Hap 10
Hap 1
Hap 13
Hap 7
Hap 2
Hap 11
Hap 18
Hap 16
Hap 5
Hap 19
Hap 6
Hap 8
Hap 17
Hap 3
Hap 9
10 samples
1 sample
BEJ
CHQ
FUJ
GAS
HEN
HUB
JIS
JIX
LIN
NIX
SHD
SIC
YUN
ZHJ

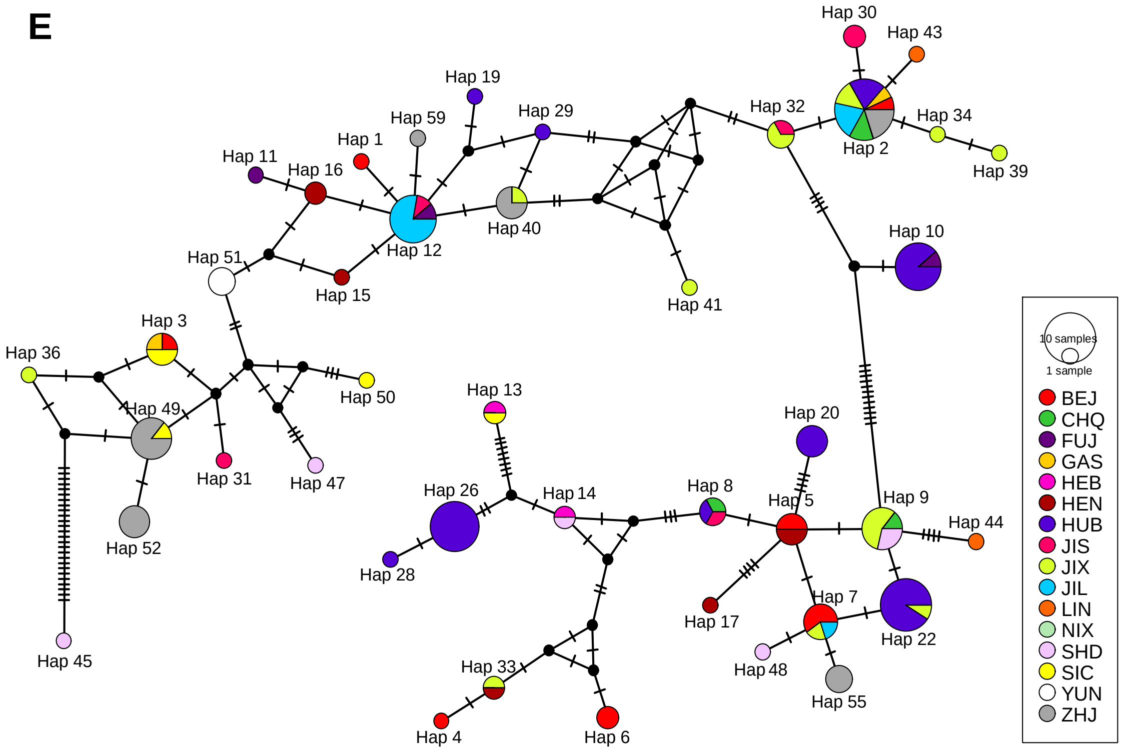

Figure S18. Median-joining (MJ) haplotype networks were analyzed to evaluate the genetic diversity in *D. eres*. **A.** translation elongation factor 1-*α* (*EF1-α*), **B.** calmodulin (*CAL*), **C.** beta-tubulin 2 (*TUB2*), **D.** histone-3 (*HIS*), and **E.** ribosomal internal transcribed spacer regions (ITS). Each circle represents a unique haplotype, and its size reflects the number of individuals expressing that haplotype. Crosshatches are indicative of the number of nucleotide differences between haplotypes. Color codes denote geographic populations. Geographic location abbreviation, BJ: Beijing; CQ: Chongqing; FJ: Fujian; GS: Gansu; HEB: Hebei; HN: Henan; HUB: Hubei; JS: Jiangsu; JX: Jiangxi; JL: Jilin; LN: Liaoning; NX: Ningxia; SD: Shandong; SC: Sichuan; YN: Yunnan; and ZJ: Zhejiang.
